# Physical activity and heat stress shape water needs in pregnant endurance athletes

**DOI:** 10.1093/emph/eoaf003

**Published:** 2025-02-04

**Authors:** Srishti Sadhir, Amanda McGrosky, Zane S Swanson, Anna Tavormina, Keri Tomechko, Herman Pontzer

**Affiliations:** Department of Evolutionary Anthropology, Duke University, Durham, NC, USA; Department of Evolutionary Anthropology, Duke University, Durham, NC, USA; Department of Biology, Elon University, Elon, NC, USA; Global Food and Water Security Program, Center for Strategic and International Studies, Washington, DC, USA; Department of Evolutionary Anthropology, Duke University, Durham, NC, USA; Duke Global Health Institute, Duke University, Durham, NC, USA; Department of Evolutionary Anthropology, Duke University, Durham, NC, USA; University of California, San Diego School of Medicine, La Jolla, CA, USA; Department of Evolutionary Anthropology, Duke University, Durham, NC, USA; Duke Global Health Institute, Duke University, Durham, NC, USA

**Keywords:** water turnover, pregnancy, physical activity, heat index, climate change

## Abstract

**Background and objectives:**

Pregnancy, heat stress, and physical activity (PA) are all known to independently increase human water requirements. We hypothesize that climate conditions and behavioral strategies interact to shape water needs in highly active pregnancies.

**Methodology:**

We recruited 20 female endurance runners who were pregnant (8–16 weeks gestational age; *n* = 13) or planning to be pregnant (*n* = 7) for an observational, prospective cohort study. At three timepoints in the study (preconception, 8–16 weeks, and 32–35 weeks), we measured water turnover (WT) using the deuterium dilution and elimination technique, PA using ActiGraph wGT3X-BT accelerometers, and heat index (HI) using historical temperature and humidity data. We also compared athletes to nonathletes from a previously published study.

**Results:**

Athletes maintained high WT from preconception through the end of pregnancy. PA was positively associated with WT among athletes for preconception and early pregnancy time periods but not for the third trimester. HI weakly moderated the relationship between PA and WT in predicting a more positive slope in hotter and more humid weather conditions. WT in athletes was higher than in nonathletes, but this difference attenuated during the third trimester, as nonathletes increased their WT.

**Conclusions and implications:**

Athletes experience higher WT with greater levels of PA, and this relationship is somewhat stronger in higher HI conditions. With the threat of climate change expected to exacerbate extreme heat conditions, evidence-based, global policies are required for particularly vulnerable populations.

## INTRODUCTION

Water needs and water turnover (WT; L/day) increase during pregnancy in step with increased daily energy expenditure and total body water (TBW; kg) to accommodate the growing fetus [1, 2]. In nonpregnant cohorts, high physical activity (PA) and high ambient temperature also increase water needs through water loss via sweating for evaporative cooling [1, 3–5]. In this paper, we consider how pregnancy, PA, and weather conditions interact to shape water needs. We examine changes to WT in pregnant athletes who train during pregnancy under varied environmental conditions. Water needs are often estimated in pregnant populations [6] but this study employs the gold-standard deuterium dilution method to measure TBW and WT.

### Water intake requirements

The National Academy of Medicine recommends daily water intake as: 3.7 l/day for males; 2.7 l/day for nonpregnant, nonlactating females; 3.0 l/day for pregnant females; and 3.8 l/day for lactating females [7]. In 458 American adults, measured WT was 3.0 l/day for males and 2.5 l/day for females [8], similar to self-reported NHANES water intake data [5]. In a global sample of 5604 adults, WT was higher: 4.3 l/day for males and 3.4 l/day for females [1]. Globally, water intake varies widely, from 1 to 10 l/day, which, in addition to age, sex, and body mass, reflects differences in environment and behavior [1, 3–5].

In pregnancy, TBW expands by 7–8 l, making up nearly 61% of total gestational weight gain (GWG), due to the modified effects of the renin-angiotensin-aldosterone and atrial natriuretic peptide pathways [2, 9]. Notably, TBW accretion, through blood plasma volume expansion, is positively correlated with neonatal birth weight and outcomes [2, 9]. Greater fluid intake is also positively associated with amniotic fluid index, which affects fetal outcomes [10]. From a clinical perspective, ensuring that water intake requirements are met during pregnancy reduces the risk of adverse outcomes in expecting mothers [2, 9, 11–16].

### Maintaining water balance in pregnancy

With various physiological and environmental challenges faced by mother and fetus, pregnancy acts as a strong selective force in determining initial survival and long-term health over the life course [17]. Due to increased water needs paired with high rates of water excretion [2], pregnant females are at greater risk of dehydration, which can negatively impact maternal and fetal health [2, 11, 14, 16, 18]. Previous research has shown total fluid intake (i.e. WT rate) increases to mitigate dehydration in pregnancy, as measured by urinary biomarkers of hydration [19]. Water needs also increase with PA [1, 3, 4, 20], and endurance athletes routinely experience acute dehydration during competitions [20, 21]. It is unknown how water intake requirements change during highly active pregnancies, especially in chronically hot environmental conditions [11]. These variables likely interacted in the human evolutionary past as pregnant females undertook active, subsistence-based lifestyles in variable environments.

In pregnant Gambian farmers, frequent heat exposure during agricultural tasks was associated with increased maternal heat exhaustion symptoms and increased maternal and fetal heat strain [15]. Participants reported behavioral changes to mitigate heat stress when possible [22]. Indeed, Yamada et al. found a smaller effect of outdoor temperature on WT in industrialized populations that are buffered by indoor climate control.

Climate change poses a particular threat to pregnancies in populations without adequate indoor climate control. Globally, 1.8–4.1 billion people lack indoor cooling solutions, many of whom undertake physically demanding occupations, even in pregnancy [23]. Current pregnancy guidelines support ≥ 150 min per week of moderate-intensity PA in mild-moderate temperatures and avoidance of high heat/humidity exposure during exercise [24–26]. However, these guidelines lack consistency, are not fully evidence-based, and are not specific about exposure time, gestational age, temperature ranges, heat acclimatization, and PA types. It is imperative to understand how both PA and environmental conditions affect water needs in pregnancy to mitigate health risks [11, 13].

### Hypothesis and predictions

We hypothesize that weather conditions and behavioral strategies interact to shape water needs in pregnant athletes:

- *Prediction 1*: Mean daily WT is greater in athletes compared to relatively sedentary nonathletes.
- *Prediction 2*: Moderate-to-vigorous PA (MVPA) and mean daily step count are independently and positively associated with WT in the preconception period and across pregnancy. The slope of this association is expected to be greater with a higher heat index (HI).
- *Prediction 3*: Irrespective of PA, there is a positive association between daytime HI and WT in the preconception period and across pregnancy.

To test these predictions, we investigated behavioral and physiological changes over pregnancy in endurance athletes living in diverse climatic regions. We also compare water needs between athlete and nonathlete pregnancies [27].

## METHODOLOGY

### Participant recruitment and study design

Female participants who were either pregnant (<16 weeks gestational age) or planning a pregnancy were recruited for an observational, prospective cohort study. We recruited participants aged 18–35 years with preconception BMI 18.5–26.0 kg/m^2^ who resided in the USA or Canada and who did not use medications that alter metabolism. Participants were required to self-report at least 300 min/week MVPA prior to conception, inclusive of >32 km/week of running and training at Tier 2: at least 3 times/week with the purpose of competing at the local level or above [28]. This research was approved by the Institutional Review Board of Duke University (Pro00108341). All participants provided informed consent upon recruitment. All study activities were conducted remotely for nationwide recruitment and study compliance with COVID-19 restrictions.

### Deuterium dilution and elimination method

We measured TBW (kg) and WT (L/day) 2–3 times (preconception, if applicable; 8–16 weeks; 32–35 weeks) using the deuterium dilution and elimination technique [29]. This method enriches the participant’s body water pool in deuterium (D), a stable, naturally occurring isotope of hydrogen. The rate of deuterium depletion was measured over a 7-day period from isotopic enrichments in the 3 postdose urine samples using the slope-intercept method. Details on this method are found in Supplementary Materials.

### PA variables

Participants continuously wore a hip-worn ActiGraph wGT3X-BT accelerometer (Actigraph, LLC) during each deuterium dilution week, including during sleep. This triaxial device measures displacement and acceleration at a 30 Hz sampling rate to quantify movement [30, 31]. Participants self-reported when they placed and removed the device. Files were downloaded from the ActiLife 6 software (Version 6.13.5, ActiGraph, LLC). Data processing took place in R [32] using *GGIR* (version 3.0.9) and *PhysicalActivity* (version 0.2.4) packages [33, 34]. Wear-time validation and data processing details can be found in Supplementary Materials. The PA outcomes were daily step counts over 7 days from the *PhysicalActivity* package and mean MVPA in 10-minute bouts over 7 days from the *GGIR* package.

### Comparative nonathlete data

We extracted TBW and WT data from a comparative study of energy requirements during pregnancy in nonathlete participants of normal BMI (19.8–26.0 kg/m^2^; *n* = 34) from Butte et al. [27]. For recruitment, participants self-reported “moderate activity,” defined as 20–30 min of moderate exercise at least three times per week [27]. Gestational age of 0 weeks was assigned as “preconception,” 22 weeks as “early pregnancy,” and 36 weeks as “third trimester” to allow for comparison with the athlete participants. Slopes of deuterium depletion (kD) and the dilution spaces of hydrogen (NH; kg) and oxygen (NO; kg) were extracted from Table 4 in Butte et al. [27] and used to calculate TBW and WT (Supplementary Materials).

### HI variable

Historical weather data was downloaded from The National Weather Service (www.wunderground.com/) based on participant zip codes. We collected daily weather metrics for the 7 days spanning the deuterium dilution week. For temperatures greater than or equal to 80°F, HI was calculated with the Rothfusz equation [35]. For temperatures below 80°F, HI was calculated with the simplified Rothfusz equation (Supplementary Materials). To ensure we captured daytime HI, we averaged HI values per day between 6:00 and 18:00 and then averaged all daily values across the 7-day deuterium dilution period.

### Statistical analysis

All data manipulation and analyses were conducted in R [32] (Supplementary Materials). We used one-way ANOVA tests and Bonferroni post hoc tests to assess potential changes in mean PA and WT between time periods.

We used random-effects linear panel regression to test for the independent effects of HI and PA on WT, as well as an interaction effect of HI*PA on WT. Gestational age and BMI were included as covariates. Models were indexed by subject and time period:


$${\text{WT}}^{∼}\text{HI}+\text{MVPA}+\text{HI*MVPA}+\text{BMI}+\text{gestational age}$$

$${\text{WT}}^{∼}\text{HI}+\text{step count}+\text{HI}*\text{step count}+\text{BMI}+\text{gestational age}$$

We used multiple linear regression to test for the independent effects of HI and PA on WT within each time period:


$${\text{WT}}^{∼}\text{HI}+\text{MVPA}+\text{BMI}+\text{gestational age}$$

$${\text{WT}}^{∼}\text{HI}+\text{step count}+\text{BMI}+\text{gestational age}$$

We compared TBW and WT results between the athletes and nonathletes using a one-way repeated measures ANOVA test (Type III), as well as an ANCOVA test (Type III) to include body mass and gestational age as covariates. To assess differences in WT between athletes and nonathletes at each time period, we employed a Bonferroni post hoc test.

## RESULTS

### Demographic summary and descriptive statistics

**Figure 1.**
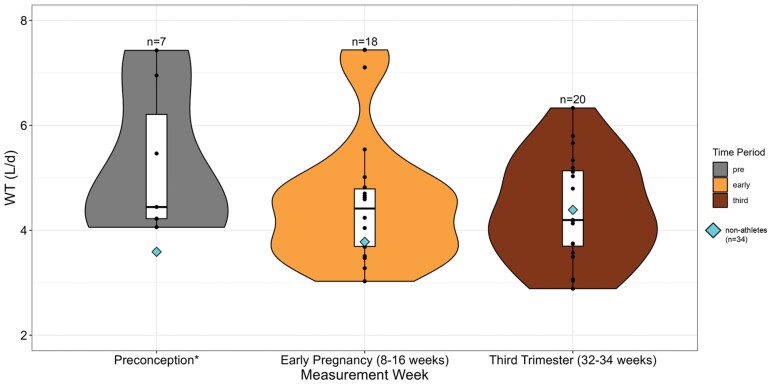
Combined violin-boxplots demonstrate no significant difference in WT across preconception, early pregnancy, and third trimester time periods in athletes (*n* = 20). Mean WT from nonathletes (*n* = 34) across the three-time points are represented by diamonds. Results from one-way ANOVA investigating WT differences between study (athletes vs. nonathletes) indicate no significant difference: *df* = 1, Sum Sq = 4.12, Mean Sq = 4.12, *F* = 3.38, *P* = .07.

A total of 20 athlete participants were included in this study. Demographic and anthropometric variables are listed in Table 1 and Supplementary Table S1. At enrollment, participants had a mean age of 32.1 ± 1.9 years. Seven participants began data collection in the preconception period, while 13 participants began data collection in early pregnancy at a mean gestational age of 15.2 ± 5.4 weeks. Participants remained in the study for 10.8 ± 3.4 months. Total GWG was 12.7 ± 3.9 kg. Mean daily WT did not differ between time periods (*F* = 1.51, *P* = .23) (Fig. 1; Table 2). Mean TBW increased over gestation (F = 6.75, p = 0.003) (Table 2).

**Table 1.** Age and anthropometric variables at enrollment, as well as total GWG in athletes (*n* = 20) and nonathletes (*n* = 34) (mean ± SD).

Variables at enrollment	Athletes (*n* = 20)	Nonathletes (*n* = 34)
Age (years)	32.1 ± 1.9	30.3 ± 4.3
Preconception body mass (kg)	59.0 ± 8.2	59.3 ± 6.0
Preconception BMI (kg/m^2^)	21.6 ± 2.4	–
Total gestational weight gain (kg)	12.7 ± 3.9	14.5 ± 4.5

SDs were not computed for the nonathlete group, since these data were drawn from published, aggregated data tables [8].

**Table 2.** PA and WT characteristics in preconception, early pregnancy, and the third trimester (mean ± SD).

Variable	Group	Preconception	Early pregnancy (8–16 weeks)	Third trimester (32–25 weeks)	ANOVA				
					*df*	Sum Sq	Mean Sq	*F* statistic	*P*-value
TBW (kg)	Athletes (*n* = 20)	35.27 ± 3.89	33.52 ± 3.68	38.08 ± 3.97	2	199.6	99.78	6.75	.003^**^
	Nonathletes (n = 34)	31.68 ± 3.90	33.97 ± 3.81	38.70 ± 4.05					
Mean daily WT (l/day)	Athletes (*n* = 20)	5.26 ± 1.41^a^	4.51 ± 1.21	4.40 ± 0.98	2	3.96	1.98	1.51	.23
	Nonathletes (n = 34)	3.59 ± 0.43	3.78 ± 0.42	4.39 ± 0.45	–	–	–	–	–
One-week running distance (km)	Athletes (*n* = 20)	48.3 ± 17.6	44.4 ± 19.5	16.7 ± 22.8	2	8036	4018	9.01	.0006^***^
One-week MVPA in 10-min bouts (min)	Athletes (*n* = 20)	461.6 ± 164^a^	410.2 ± 163.8	402.4 ± 380.0	2	108 844	54 422	1.61	.21
One-week mean daily step count	Athletes (*n* = 20)	14 075 ± 3795^a^	11 562 ± 3155	9226 ± 3962	2	134 480 574	67 240 287	5.10	.01^*^
Mean daytime HI (°F)	Athletes (*n* = 20)	58.6 ± 26.5	64.6 ± 18.6	64.8 ± 16.8	2	15 442	109.7	0.30	.74

^a^Preconception athlete data based on *n* = 7 sample.

^*^ *P* ≤ .05,

^**^ *P* < .01,

^***^ *P* < .001.

Results from ANOVA were not computed for the nonathlete group, since these data were drawn from published, aggregated data tables. Results from Bonferroni post hoc tests can be found in Supplementary Materials (Supplementary Tables S2–S7).

Data on PA are summarized in Table 2. There were 315 days of accelerometry with a 92.7% adherence, yielding 292 wear days with an average of 6.5 days per participant per measurement period. For participants recruited in the preconception period (*n* = 7), step count was 14 075 ± 3795 steps/day, and MVPA was 461.6 ± 164.5 min/week. Preconception running distance was 48.3 ± 17.6 km/week. Weekly running distance decreased from early pregnancy (44.4 ± 19.5 km/week) to the third trimester (16.7 ± 22.8 km/week) (*F* = 9.01, *P* = .0006), with 11 participants stopping their running activities altogether by the third trimester. Participants maintained MVPA (461.6 ± 164.5 min/week in preconception, 410.2 ± 163.8 min/week in early pregnancy, and 402.4 ± 380.0 min/week in the third trimester) (*F* = 1.61, *P* = .21), though the range and variability in MVPA were greater during the third trimester (SD: 163.8 vs. 380.0 min/week). Participants reported more low-impact endurance activities as alternatives to running in late pregnancy to mitigate pain, soreness, and changes to gait and pacing. Alternative activities included swimming, cycling, and brisk walking. Daily step count was reduced from preconception (14 075 ± 3795 steps/day) to the third trimester (9226 ± 3962 steps/day) (*F* = 5.10, *P* = .01).

### Linear panel regression analysis of the effects of PA, HI, and PA*HI on WT

There was no relationship between MVPA and WT (*β* = −0.0026, *P* = .30), nor between HI and WT (*β* = −0.021, *P* = .16) (Table 3). There was a positive, nonsignificant trend in the interaction effect of MVPA*HI on WT (*β* = 0.000067, *P* = .05). For the step count outcome, there was no relationship between daily step count and WT (*β* = −0.000046, *P* = .65), nor between HI and WT (*β* = −0.025, *P* = .18) (Table 3). There was no relationship in the interaction effect of step count*HI on WT (*β* = 0.0000026, *P* = .10). By visualizing the postestimation plots of the panel regression models (Supplementary Fig. S1), we found possible evidence of a more positive relationship (slope) between MVPA and WT, as well as step count and WT, as HI increased.

**Table 3.** Random-effects linear panel regression models to test for the independent effects of HI and PA on WT, as well as an interaction effect of HI*PA on WT, in panel (longitudinal) data for athletes only.

WT ~ HI + MVPA + HI*MVPA + BMI + gestational age			WT ~ HI + step count + HI*step count + BMI + gestational age		
Covariate	β Coefficient (SE)	*P*-value	Covariate	β Coefficient (SE)	*P*-value
(intercept)	3.28 (1.84)	.08	(intercept)	3.36 (1.97)	.08
HI	−0.021 (0.015)	.16	HI	−0.025 (0.018)	.18
MVPA	−0.0026 (0.0024)	.30	Step count	−0.000046 (0.00010)	.65
BMI	0.089 (0.075)	.23	BMI	0.061 (0.071)	.86
Gestational age	−0.0090 (0.011)	.41	Gestational age	−0.0023 (0.016)	.99
HI*MVPA	0.000067^*^ (0.000036)	.05^*^	HI*step count	0.0000026 (0.0000015)	.10
Total Sum of Squares	13.39		Total Sum of Squares	14.90	
Residual Sum of Squares	13.60		Residual Sum of Squares	13.96	
R-Squared	0.09		R-Squared	0.12	

^*^ *P* ≤ .05; ^**^P < .01; ^***^P < .001.

Models are indexed by participant and measurement time period. The interaction effect HI*MVPA was the only marginally significant factor in the models.

### Multiple linear regression analysis of the independent effects of MVPA and HI on WT during each time period

**Figure 2.**
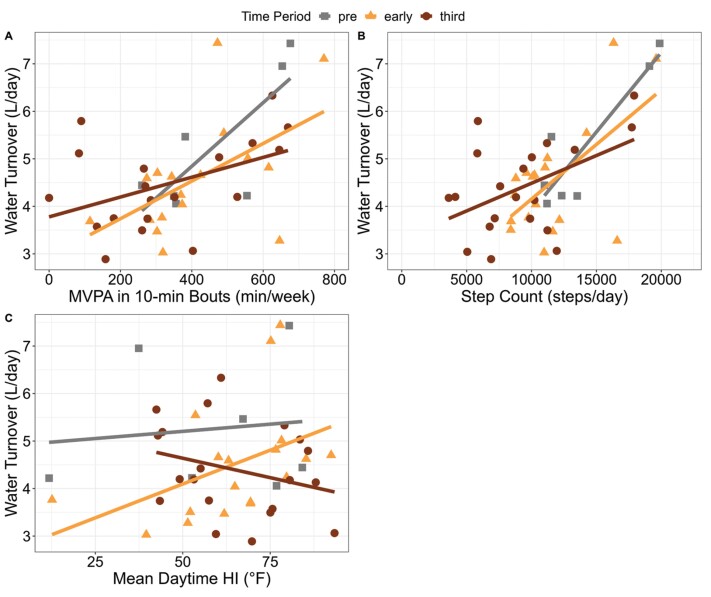
Relationship between environmental factors and WT in athletes. (a) MVPA in 10-minute bouts positively predicts WT in early pregnancy only. (b) Step count positive predicts WT in preconception and early pregnancy only. (c) There is no relationship between mean daytime HI and WT at any time period during preconception and pregnancy.

In the preconception period, there was no relationship between MVPA and WT (*β* = 0.052, *P* = .54), nor between HI and WT (*β* = 0.017, *P* = .81) (Fig. 2a and c; Supplementary Table S8). In early pregnancy, there was a significant, positive relationship between MVPA and WT (*β* = 0.0043, *P* = .02), where there was a 0.13 l increase in daily WT with every 30-min weekly increase in MVPA (Fig. 2a; Supplementary Table S9). There was no relationship between HI and WT (*β* = 0.016, *P* = .27) (Fig. 2c; Supplementary Table S9). In the third trimester, there was no relationship between MVPA and WT (*β* = 0.001, *P* = .38), nor between HI and WT (*β* = −0.018, *P* = .14) (Fig. 2a and c; Supplementary Table S10).

### Multiple linear regression analysis of the independent effects of step count and HI on WT during each time period

In the preconception period, there was a significant, positive relationship between step count and WT (*β* = 0.00034, *P* = .02), where there was a 0.34 l increase in daily WT with each 1000-step daily increase (Fig. 2b; Supplementary Table S8). There was no relationship between HI and WT (*β* = 0.019, *P* = .20) (Fig. 2c; Supplementary Table S8). In early pregnancy, there was a significant, positive relationship between step count and WT (*β* = 0.00025, *P* = .004), where there was a 0.25 L increase in daily WT with every 1,000-step daily increase (Fig. 2b; Supplementary Table S9). There was a positive, nonsignificant trend between HI and WT (*β* = 0.023, *P* = .07) (Fig. 2c; Supplementary Table S9). In the third trimester, there was no relationship between step count and WT (β=0.00005, p = 0.43), nor between HI and WT (*β* = −0.019, *P* = .13) (Fig. 2b and c; Supplementary Table S10).

### Comparative analysis between athletes and nonathletes

Mean TBW for nonathletes was 31.68 ± 3.90 kg in preconception, 33.97 ± 3.81 kg in early pregnancy, and 38.70 ± 4.05 kg in the third trimester (Table 2). Mean WT for nonathletes was 3.59 ± 0.43 l/day in preconception, 3.78 ± 0.42 l/day in early pregnancy, and 4.39 ± 0.45 l/day in the third trimester (Table 2).

Athletes did not have greater TBW than nonathletes (*F* = 2.87, *P* = .09). However, when accounting for body mass and gestational age, group was a significant predictor of TBW (*F* = 6.81, *P* = .01) (Supplementary Table S11). Body mass (*F* = 239.11, *P* < .0001) and gestational age (*F* = 141.61, *P* < .0001) were significant predictors of TBW, with greater effects than group designation (Supplementary Table S11). TBW was only greater in athletes compared to nonathletes in preconception (*P* < .0001) (Supplementary Table S12). There was no difference in TBW between groups in early pregnancy (*P* = .48) and in the third trimester (*P* = .36) (Supplementary Table S12).

Athletes had greater daily WT overall compared to nonathletes (*F* = 27.29, *P* < .0001) (Supplementary Table S13). When accounting for body mass and gestational age, group was still a significant predictor of WT (*F* = 17.54, *P* < .0001) (Supplementary Table S13). Body mass (*F* = 3.66, *P* = .06) and gestational age (*F* = 3.35, *P* = .07) were marginal predictors of WT (Supplementary Table S13). The difference in WT between athletes and nonathletes was driven by preconception (*P* < .0001) and early pregnancy (*P* < .0001) time periods (Supplementary Table S14). There was no difference in WT between groups in the third trimester (*P* = .99) (Supplementary Table S14).

## DISCUSSION

Our results somewhat support the hypothesis that weather conditions and behavioral strategies interact to shape water needs in pregnant athletes. While athletes did not reduce MVPA from preconception to the third trimester, daily step count was significantly reduced. Step count includes habitual activities throughout the 24-h cycle, whereas MVPA may represent more intentional PA bouts in this population [36–38]. Athletes accommodated MVPA by reducing nonintentional PA throughout the day, or by moving to lower-impact, lower-intensity activities. In doing so, WT did not increase throughout pregnancy. Since HI did not predict WT, athletes may have moved indoors, shifted activity patterns to different times of the day, or moved to more amenable locations for PA to reduce sweating and water intake needs. Despite the role of behavioral modification, we were able to detect a slight positive interaction effect of PA and HI on water requirements across gestation.

Expansion of TBW occurred similarly in both athlete and non-athlete pregnancies, given similar body sizes between groups, so WT rate is the primary physiological strategy to deal with water stress. Athletes in preconception had a higher WT (5.26 l/day) than nonathlete participants (3.59 l/day), a global sample of nonpregnant females (3.4 l/day), and an American sample of nonpregnant females (2.5 l/day), reflecting the additional water needs with high PA [1, 8]. Both the athlete (4.40 l/day) and nonathlete (4.39 l/day) cohorts had higher WT in the third trimester compared to the global and American samples [1, 8], reflecting the additional water needs associated with pregnancy.

We are unsure of PA differences between the athletes and nonathletes, since accelerometry was not routinely employed in studies until the 2010s [39]. However, studies of pregnancy in other nonathlete cohorts report low MVPA, particularly in the third trimester [40–46]. A PA recruitment criterion was included for the non-athlete comparative sample, which was lower than athlete recruitment (*Methodology*). Given the prevalence of overreporting bias in survey-based PA measurements [47], preconception PA in the nonathlete sample was likely lower than the athlete sample in this study.

Pregnant people across the world face multiple stressors to water balance, including lack of indoor climate control; exposure to hot, water-scarce, and nonpotable water environments; and high workloads [15, 23]. While pregnant people are told to avoid high heat and humidity during PA, not enough data exists to establish thresholds [24–26]. Our results suggest that the current water intake guidelines that exist are too low for highly active pregnant populations experiencing high heat stress. With the threat of climate change expected to exacerbate extreme heat conditions, evidence-based policies are required to protect particularly vulnerable populations.

When comparing humans with other apes, Pontzer and colleagues [48] found that humans have evolved better water conservation strategies, despite having a higher sweating capacity through high eccrine gland density [49, 50]. These strategies may include the development of external noses for water recovery [48] and lower hair density to reduce sweating [49, 50]. It is also unknown how water requirements differ in pregnancy between ape species, since interspecies TBW expansion, urine concentrating abilities, and other pregnancy traits may be variable. In addition, social buffering of water stress may be more prevalent in humans, as interdependence within social groups (e.g. cooperative breeding) may better allow pregnant individuals to seek amenable environmental conditions and reduce PA [51]. Conversely, females of other ape species often undertake pregnancy without assistance from group members [51].

### Limitations

We were unable to account for specific microclimates experienced by athletes. Indoor heat stress is an important driver of global heat-related morbidity and mortality [52]. Athletes were likely buffered by indoor climate control, which has been found in other higher-income populations [1]. As a result of higher-income status, this population may experience better economic/social buffering to accommodate PA during pregnancy, even in climates with high heat stress, without suffering negative health outcomes. Thus, athletes in this study are not representative of global climate experience across all pregnant populations.

This study relied on PA measured by accelerometry, which tracks acceleration-based activities. For this reason, we restricted recruitment of athletes who predominantly undertake other modes of exercise, like weight training. It is likely that we did not capture stationary activities through accelerometry, which still contribute to overall water needs.

## CONCLUSION

We report on water requirements for pregnant endurance athletes, which were higher than nonathletes but attenuated in the third trimester. PA was positively associated with WT among athletes for preconception and early pregnancy but not for the third trimester. HI weakly predicted a more positive slope in hotter, more humid weather conditions. Yet, pregnant athletes overall had good control of their environment to mitigate the risk of extreme dehydration, with no adverse outcomes reported [2, 14, 16]. Adverse pregnancy outcomes are expected to increase with climate change. To better understand dynamic water needs, a greater diversity of human populations and nonhuman primate species is required for study.

## Supplementary Material

eoaf003_suppl_Supplementary_Figure_S1

eoaf003_suppl_Supplementary_Material

## Data Availability

The de-identified data that support the findings of this study are available the OSF project repository (https://doi.org/10.17605/OSF.IO/4SVAP).

## References

[1] Yamada Y , Zhang X, Henderson MET et al; International Atomic Energy Agency (IAEA) Doubly Labeled Water (DLW) Database Consortium§. Variation in human water turnover associated with environmental and lifestyle factors. Science 2022;378:909–15. 36423296 10.1126/science.abm8668 PMC9764345

[2] Theunissen IM , Parer JT. Fluid and electrolytes in pregnancy. Clin Obstet Gynecol 1994;37:3–15. 8194213 10.1097/00003081-199403000-00005

[3] Shimamoto H , Komiya S. The turnover of body water as an indicator of health. J Physiol Anthropol Appl Hum Sci 2000;19:207–12. 10.2114/jpa.19.207 11155349

[4] Swanson ZS , Pontzer H. Water turnover among human populations: effects of environment and lifestyle. Am J Human Biol 2020;32:e23365. 31782865 10.1002/ajhb.23365

[5] Rosinger AY. Biobehavioral variation in human water needs: how adaptations, early life environments, and the life course affect body water homeostasis. Am J Human Biol 2020;32:e23338. 31631450 10.1002/ajhb.23338

[6] Armstrong LE , Johnson EC. Water intake, water balance, and the elusive daily water requirement. Nutrients 2018;10:1928. 30563134 10.3390/nu10121928 PMC6315424

[7] Institute of Medicine (U.S.). Panel on dietary reference intakes for electrolytes and water. In Dietary Reference Intakes for Water, Potassium, Sodium, Chloride, and Sulfate. Washington, DC: The National Academies Press, 2004.

[8] Raman A , Schoeller DA, Subar AF et al Water turnover in 458 American adults 40-79 yr of age. Am J Physiol Renal Physiol 2004;286:F394–401. 14600032 10.1152/ajprenal.00295.2003

[9] Rasmussen KM , Yaktine AL, Institute of Medicine (U.S.) (eds). Weight Gain during Pregnancy: Reexamining the Guidelines. Washington, DC: National Academies Press, 2009. 20669500

[10] Song Y , Zhang F, Lin G et al A study of the fluid intake, hydration status, and health effects among pregnant women in their second trimester in china: a cross-sectional study. Nutrients 2023;15:1739. 37049579 10.3390/nu15071739 PMC10096982

[11] Samuels L , Nakstad B, Roos N et al Physiological mechanisms of the impact of heat during pregnancy and the clinical implications: review of the evidence from an expert group meeting. Int J Biometeorol 2022;66:1505–13. 35554684 10.1007/s00484-022-02301-6 PMC9300488

[12] Intergovernmental Panel on Climate Change (IPCC) (ed). Health, wellbeing and the changing structure of communities. In Climate Change 2022 – Impacts, Adaptation and Vulnerability: Working Group II Contribution to the Sixth Assessment Report of the Intergovernmental Panel on Climate Change. Cambridge: Cambridge University Press, 2023, 1041–170.

[13] Jiao A , Sun Y, Avila C et al Analysis of heat exposure during pregnancy and severe maternal morbidity. JAMA Net Open 2023;6:e2332780. 10.1001/jamanetworkopen.2023.32780 PMC10485728 37676659

[14] El-Sharkawy AM , Sahota O, Lobo DN. Acute and chronic effects of hydration status on health. Nutr Rev 2015;73:97–109. 26290295 10.1093/nutrit/nuv038

[15] Bonell A , Sonko B, Badjie J et al Environmental heat stress on maternal physiology and fetal blood flow in pregnant subsistence farmers in the Gambia, west Africa: an observational cohort study. Lancet Planet Health 2022;6:e968–76. 36495891 10.1016/S2542-5196(22)00242-X PMC9756110

[16] Pazhayattil GS , Rastegar A, Brewster UC. Approach to the diagnosis and treatment of hyponatremia in pregnancy. Am J Kidney Dis 2015;65:623–7. 25542410 10.1053/j.ajkd.2014.09.027

[17] Brown EA , Ruvolo M, Sabeti PC. Many ways to die, one way to arrive: how selection acts through pregnancy. Trends Genet 2013;29:585–92. 23566676 10.1016/j.tig.2013.03.001

[18] Rosinger AY , Bethancourt HJ, Pauley AM et al Variation in urine osmolality throughout pregnancy: a longitudinal, randomized-control trial among women with overweight and obesity. Eur J Nutr 2022;61:127–40. 34218315 10.1007/s00394-021-02616-x PMC8720908

[19] McKenzie AL , Perrier ET, Guelinckx I et al Relationships between hydration biomarkers and total fluid intake in pregnant and lactating women. Eur J Nutr 2017;56:2161–70. 27519184 10.1007/s00394-016-1256-3 PMC5579181

[20] Popkin BM , D’Anci KE, Rosenberg IH. Water, hydration, and health. Nutr Rev 2010;68:439–58. 20646222 10.1111/j.1753-4887.2010.00304.x PMC2908954

[21] Beis LY , Wright-Whyte M, Fudge B et al Drinking behaviors of elite male runners during marathon competition. Clin J Sport Med 2012;22:254–61. 22450589 10.1097/JSM.0b013e31824a55d7

[22] Spencer S , Samateh T, Wabnitz K et al The challenges of working in the heat whilst pregnant: insights from Gambian women farmers in the face of climate change. Front Public Health 2022;10:1505–13. 10.3389/fpubh.2022.785254 PMC8883819 35237548

[23] Mastrucci A , Byers E, Pachauri S et al Improving the SDG energy poverty targets: Residential cooling needs in the Global South. Energy Build 2019;186:405–15.

[24] Mottola MF , Davenport MH, Ruchat S-M et al 2019 Canadian guideline for physical activity throughout pregnancy. Br J Sports Med 2018;52:1339–46. 30337460 10.1136/bjsports-2018-100056

[25] Mottola MF , Davenport MH, Ruchat S-M et al No. 367-2019 Canadian guideline for physical activity throughout pregnancy. J Obstetrics Gynaecol Canada 2018;40:1528–37. 10.1016/j.jogc.2018.07.001 30297272

[26] Evenson KR , Barakat R, Brown WJ et al Guidelines for physical activity during pregnancy: comparisons from around the world. Am J Lifestyle Med 2014;8:102–21. 25346651 10.1177/1559827613498204 PMC4206837

[27] Butte NF , Wong WW, Treuth MS et al Energy requirements during pregnancy based on total energy expenditure and energy deposition. Am J Clin Nutr 2004;79:1078–87. 15159239 10.1093/ajcn/79.6.1078

[28] McKay AKA , Stellingwerff T, Smith ES et al Defining training and performance caliber: a participant classification framework. Int J Sports Physiol Perform 2022;1:1–15. 10.1123/ijspp.2021-0451 34965513

[29] International Atomic Energy Agency. Assessment of body composition and total energy expenditure in humans using stable isotope techniques. In IAEA Human Health Series. Vienna, Austria: International Atomic Energy Agency, 2009, 3.

[30] van Hees VT , Renström F, Wright A et al Estimation of daily energy expenditure in pregnant and non-pregnant women using a wrist-worn tri-axial accelerometer. PLoS One 2011;6:e22922. 21829556 10.1371/journal.pone.0022922 PMC3146494

[31] Santos-Lozano A , Santín-Medeiros F, Cardon G et al Actigraph GT3X: validation and determination of physical activity intensity cut points. Int J Sports Med 2013;34:975–82. 23700330 10.1055/s-0033-1337945

[32] R Core Team. *R: A Language and Environment for Statistical Computing*. 2024.

[33] Choi L , Beck C, Liu Z et al *Package “PhysicalActivity.”* 2021.

[34] Migueles JH , Rowlands AV, Huber F et al GGIR: a research community–driven open source r package for generating physical activity and sleep outcomes from multi-day raw accelerometer data. J Measurem Phys Behav 2019;2:188–96.

[35] Rothfusz LP. The Heat Index “Equation” (or, More Than You Ever Wanted to Know About Heat Index). National Weather Service Technical Attachment. Forth Worth, Texas, USA, 1990.

[36] Kraus WE , Janz KF, Powell KE et al; 2018 Physical Activity Guidelines Advisory Committee*. Daily step counts for measuring physical activity exposure and its relation to health. Med Sci Sports Exerc 2019;51:1206–12. 31095077 10.1249/MSS.0000000000001932 PMC6527133

[37] Beenackers MA , Kamphuis CB, Giskes K et al Socioeconomic inequalities in occupational, leisure-time, and transport related physical activity among European adults: a systematic review. Int J Behav Nutr Phy Activ 2012;9:116. 10.1186/1479-5868-9-116 PMC3491027 22992350

[38] Powell LM , Slater S, Chaloupka FJ. The relationship between community physical activity settings and race, ethnicity and socioeconomic status. Evi-Based Preven Med 2004;1:135–44.

[39] Troiano RP , McClain JJ, Brychta RJ et al Evolution of accelerometer methods for physical activity research. Br J Sports Med 2014;48:1019–23. 24782483 10.1136/bjsports-2014-093546 PMC4141534

[40] Andersen MB , Ostenfeld EB, Fuglsang J et al Maternal prepregnancy body mass index and physical activity during pregnancy assessed by accelerometer. Am J Obstetrics Gynecol MFM 2020;2:100182. 10.1016/j.ajogmf.2020.100182 33345908

[41] Barone Gibbs B , Jones MA, Jakicic JM et al Objectively-measured sedentary behavior and physical activity across three trimesters of pregnancy: the monitoring movement and health (MoM Health) study. J Phys Act Health 2021;18:254–61. 33508775 10.1123/jpah.2020-0398 PMC8054065

[42] Hesketh KR , Evenson KR, Stroo M et al Physical activity and sedentary behavior during pregnancy and postpartum, measured using hip and wrist-worn accelerometers. Preven Med Rep 2018;10:337–45. 10.1016/j.pmedr.2018.04.012 PMC5984239 29868389

[43] Löf M. Physical activity pattern and activity energy expenditure in healthy pregnant and non-pregnant Swedish women. Eur J Clin Nutr 2011;65:1295–301. 21792212 10.1038/ejcn.2011.129

[44] Sandborg J , Migueles JH, Söderström E et al Physical activity, body composition, and cardiometabolic health during pregnancy: a compositional data approach. Med Sci Sports Exer 2022;54:2054–63. 10.1249/MSS.0000000000002996 PMC9671591 36069838

[45] Rousham EK , Clarke PE, Gross H. Significant changes in physical activity among pregnant women in the UK as assessed by accelerometry and self-reported activity. Eur J Clin Nutr 2006;60:393–400. 16306930 10.1038/sj.ejcn.1602329

[46] Vietheer A , Kiserud T, Lie RT et al Sleep and physical activity from before conception to the end of pregnancy in healthy women: a longitudinal actigraphy study. Sleep Med 2021;83:89–98. 33991895 10.1016/j.sleep.2021.04.028

[47] Rzewnicki R , Auweele YV, Bourdeaudhuij ID. Addressing overreporting on the International Physical Activity Questionnaire (IPAQ) telephone survey with a population sample. Public Health Nutr 2003;6:299–305. 12740079 10.1079/PHN2002427

[48] Pontzer H , Brown MH, Wood BM et al Evolution of water conservation in humans. Curr Biol 2021;31:1804–10.e5. 33675699 10.1016/j.cub.2021.02.045

[49] Aldea D , Atsuta Y, Kokalari B et al Repeated mutation of a developmental enhancer contributed to human thermoregulatory evolution. Proc Natl Acad Sci USA 2021;118:e2021722118. 33850016 10.1073/pnas.2021722118 PMC8072367

[50] Kamberov YG , Guhan SM, DeMarchis A et al Comparative evidence for the independent evolution of hair and sweat gland traits in primates. J Hum Evol 2018;125:99–105. 30502901 10.1016/j.jhevol.2018.10.008 PMC6289065

[51] Hrdy SB. Variable postpartum responsiveness among humans and other primates with ‘cooperative breeding’: a comparative and evolutionary perspective. Horm Behav 2016;77:272–83. 26518662 10.1016/j.yhbeh.2015.10.016

[52] Hampo CC , Schinasi LH, Hoque S. Surviving indoor heat stress in United States: a comprehensive review exploring the impact of overheating on the thermal comfort, health, and social economic factors of occupants. Heliyon 2024;10:e25801. doi: https://doi.org/10.1016/j.heliyon.2024.e25801 38371979 PMC10873744

