# Supplementary figures and images for: Physical activity and heat stress shape water needs in pregnant endurance athletes
Source: Evol Med Public Health. 2025 Feb 4;13(1):25–34. doi: 10.1093/emph/eoaf003 (PMC11879205; doi:10.1093/emph/eoaf003)

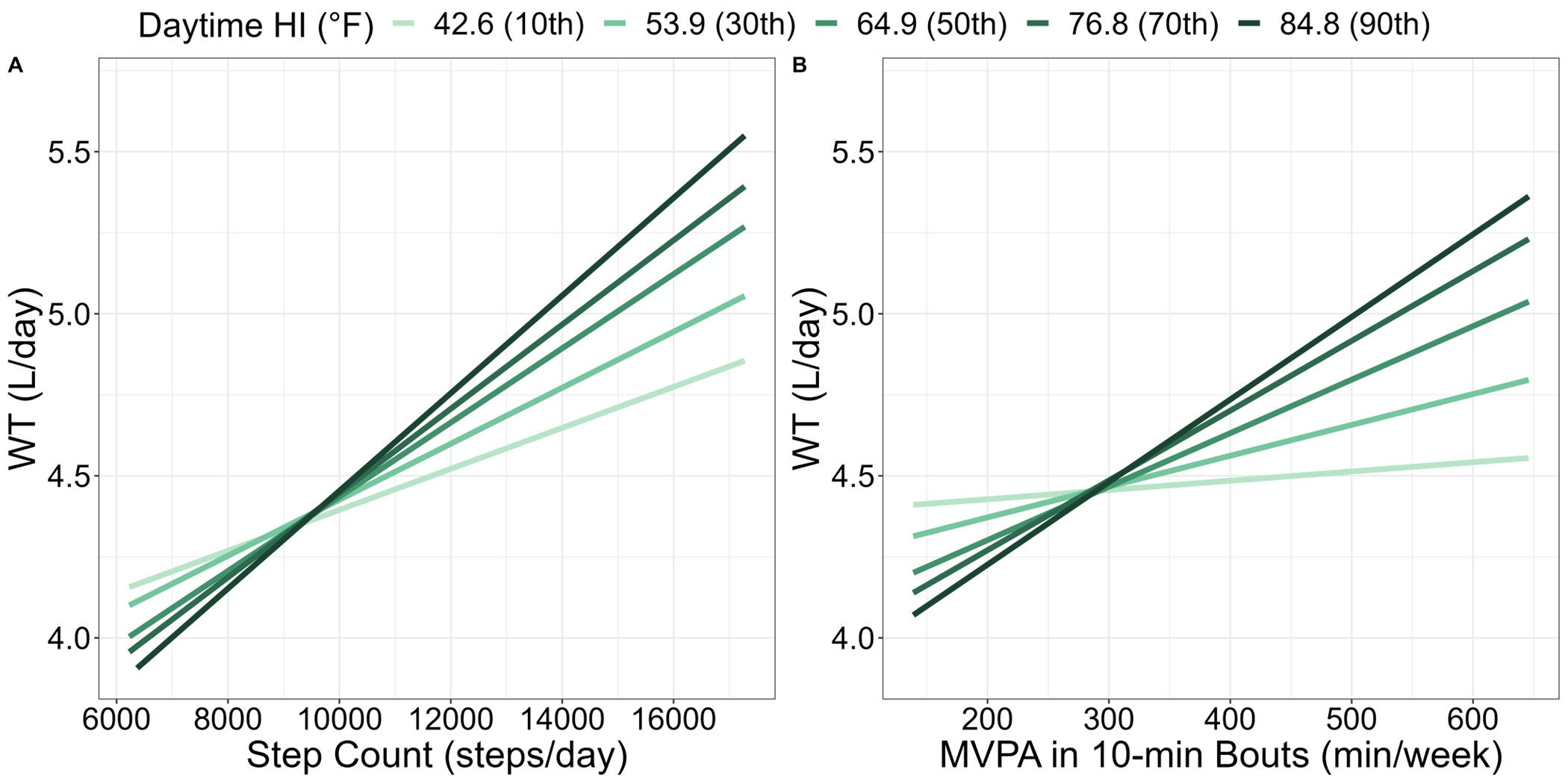

Supplement: eoaf003_suppl_Supplementary_Figure_S1 [file eoaf003_suppl_supplementary_figure_s1.jpeg]
