# Supplementary material for: Physical activity and heat stress shape water needs in pregnant endurance athletes
Source: Evol Med Public Health. 2025 Feb 4;13(1):25–34. doi: 10.1093/emph/eoaf003 (PMC11879205; doi:10.1093/emph/eoaf003)
Supplement: eoaf003_suppl_Supplementary_Material [file eoaf003_suppl_supplementary_material.docx]

**SUPPLEMENTARY MATERIALS**

***Physical activity and heat stress shape water needs in pregnant endurance athletes***

Srishti Sadhir, Amanda McGrosky, Zane S. Swanson, Anna Tavormina, Keri Tomechko, Herman Pontzer

**METHODOLOGY**

*Deuterium Dilution & Elimination Method*

Deuterium (D) is a stable and naturally occurring nonradioactive isotope found in the environment and in human body water at low levels. Ingestion is safe and non-invasive and poses no risk to humans [1]. This method has been previously employed in studies of pregnancy, lactation, and infancy [2–10]. The dose amount is calculated based on body weight, regardless of reproductive status: 1.25 g per kg body mass of water enriched to 6% D_2_-O (Sigma-Aldrich).

Before dosing, participants provided a urine sample of 5 ml to establish baseline isotopic values for comparison to enrichment values. At dosing, participants rinsed the dose bottle multiple times to ensure all deuterium is consumed. Participants then collected urine samples (5 ml each) at 6 hours, 3 days, and 7 days post-dose, stored in freezer bags within a personal freezer until shipping to the Pontzer Lab. In the lab, samples were filtered at 30 kDa in 6ml centrifuge tubes (Vivaspin 6) to separate water content from precipitate and analyzed through laser absorption mass spectrometry (ABB Systems ICOS) for D enrichment. We averaged 2-3 analyzer runs per measurement period: 4-6 sets of sample injections, with 2 sets per file. We selected runs with NH/NO ratios between 0.990-1.080 to be included in WT calculations.

We calculated TBW using the dilution spaces of hydrogen (NH; moles) and oxygen (NO; moles) [11]:

**TBW_kg_ = (NH_moles_ / 1.007+ NO_moles_ / 1.043) / 2 x 0.01802**

We calculated WT using the slope of deuterium depletion (kD) and the dilution space of hydrogen (NH; moles) [11]:

**WT_L/day_ = |(kD × 1.043 × NH_moles_ / 0.99) × 18.02 / 1000|**

For the comparative non-athlete data from Butte et al. [12], we used the slopes of deuterium depletion (kD) and the dilution spaces of hydrogen (NH; kg) and oxygen (NO; kg) to calculate TBW and WT. The NH and NO values were converted from kilograms to moles using the molar mass of water:

**NH_moles_ = NH_kg_ / 0.01802**

**NO_moles_ = NO_kg_ / 0.01802**

We calculated WT (L/day) [37], where kD is the negative slope of deuterium depletion over time, and NH (moles) is the dilution space of hydrogen:

**WT_L/day_ = |(kD × 1.043 × NH_moles_ / 0.99) × 18.02 / 1000|**

We also calculated TBW (kg) [37], where NH (moles) is the dilution space of hydrogen and NO (moles) is the dilution space of oxygen in the body:

**TBW_kg_ = (NH_moles_ / 1.007+ NO_moles_ / 1.043) / 2 x 0.01802**

We used Gaussian error propagation to calculate SD for TBW and WT, since components of the above equations were aggregated as means and SDs in the Butte et al. [8] article.

*Heat Index Calculation*

For temperatures greater than or equal to 80°F, HI was calculated with the Rothfusz [38] equation using temperature (T; °F) and relative humidity (RH; %):

**HI = -42.379 + (2.04901523 x T) + (10.14333127 x RH) – (0.22475541 x T x RH) – (0.00683783 x T x T) – (0.05481717 x RH x RH) + (0.00122874 x T x T x RH) + (0.00085282 x T x RH x RH) – (0.00000199 x T x T x RH x RH)**

For temperatures below 80°F, HI was calculated with the simplified Rothfusz equation (<https://www.wpc.ncep.noaa.gov/html/heatindex_equation.shtml>):

**HI = 0.5 x {T + 61.0 + [(T-68.0) x 1.2] + (RH x 0.094)}**

*PhysicalActivity Package* [13]

The converted .AGD files (1-second epoch) were binned into 1-minute-epoch count .CSV files. Based on self-reported placement and removal, we constrained the data files to each participant’s wear period. Then, based on Withings sleep metrics, we coded sleep and wake times over the total wear period. Mean daily step counts over 7 days were analyzed for the full 24-hour period.

*GGIR Package Shell Function Configuration* [14]

The raw .GT3X files were applied to the *GGIR* package in R. The *GGIR* shell function was modified to fit the parameters of our study design. Acceleration-based cut-offs for PA intensity derive from published data on hip-worn ActiGraph devices for adults (age range: 21-61 years) [15,16]. An advanced sleep log was created using the Withings Pulse HR sleep data. For days the Withings device was not worn, wake and sleep time were estimated using sleep patterns of preceding or succeeding days. Raw .GT3X files (recorded in 30 Hz) were imported into GGIR shell function. Shell function specifications are described in package vignette ([https://cran.r-project.org/web/packages/GGIR/vignettes/GGIRParameters.html#](https://cran.r-project.org/web/packages/GGIR/vignettes/GGIRParameters.html)), and all specifications were left on default settings except the following:

- Time zones were specified based on participants’ locations.
- sensor.location = "hip" (The device was hip-worn.)
- do.anglex = TRUE (Triaxial accelerometers were used.)
- do.angley = TRUE (Triaxial accelerometers were used.)
- printsummary = TRUE (This specification shows a summary of the calibration procedure.)
- includedaycrit = 10 (Some participants were wearing the accelerometer only during waking hours and not early morning or late evenings, so we want to capture as many days as possible by specifying a 10-hour day.)
- qwindow = c(6, 24) (This specification allows us to capture the fullest waking day length i.e., 6am to midnight, in addition to the 24-hour cycle.)
- max_calendar_days = 0 (Participants had variable number of days for which the accelerometer was worn.)
- iglevels = c(seq(0,4000,by=25), 8000) (We calculated intensity gradients following [17])
- HASPT.algo = "HorAngle" (The sensor location was at the hip.)
- loglocation specified based on file location for the advanced sleep log using Withings sleep data.
- sleepwindowType = "TimeInBed" (This specification aligns with HASPT.algo = "HorAngle" from above.)
- outliers.only = TRUE
- criterror = 4
- mvpadur = c(1, 5, 10) (We specified MVPA to be calculated in 1-minute, 5-minute, and 10-minute bouts.)
- threshold.lig = c(47.4) (Accelerometry-based thresholds derive from [15,16].)
- threshold.mod = c(69.1) (Accelerometry-based thresholds derive from [15,16].)
- threshold.vig = c(258.7) (Accelerometry-based thresholds derive from [15,16].)
- timewindow = c("WW") (This specification allowed for visualization of waking period only.)

*GGIR Outcome: MVPA in 10-min Bouts for Waking Period Only* [14]

We calculated the average number of valid wear days, defined as 10 or more hours of waking wear time and where non-wear time was less than 30% of this time. We extracted the “dur_day_MVPA_bts_10_min” variable from the Part 5: day summary .CSV file. We then calculated mean daily MVPA in 10-min bouts, since some participants didn’t have 7 or more valid wear days. Mean daily MVPA was multiply by 7 to get MVPA in minutes per week.

*Statistical Analysis: Model Selection & Specifications*

For all regression models, we determined the best fit based on data residual (error) distribution. We examined normality and heteroscedasticity in residuals using several methods. QQ plots, density plots, and ECDF plots all indicated approximately normal distribution. Results from the Shapiro-Wilk test of normality [18] aligned with the ECDF plot for all models. Results from the Breusch-Pagan test of heteroscedasticity [19] indicated no heteroscedasticity at significance level of α=0.05. Thus, the ordinary least squares (Gaussian distribution) method was chosen for statistical modeling and robust SEs were not computed. For all one-way ANOVA tests, results from Levene's test for homogeneity of variance [20] indicated no difference in variance across groups at significance level of α=0.05. For panel regression models, we did not detect autocorrelation using the Breusch-Godfrey test [21,22] at significance level of α=0.05. The random-effects designation was chosen for panel regression models based on the results of the Durbin-Wu-Hausman test [23–26] (p>0.05), which tests if individual errors are correlated with the predictors.

**RESULTS**

**Table S1.** Demographic and socioeconomic variables at enrollment in athletes only.

| Variables at Enrollment | Mean ± SD, or n (%) |
| --- | --- |
| Ethnicity |  |
| *Not Hispanic or Latino* | 19 (95%) |
| *Hispanic or Latino* | 1 (5%) |
| Race |  |
| *White* | 19 (95%) |
| *More than one race* | 1 (5%) |
| *Other** | 0 (0%) |
| Geographic region  (U.S. Census Bureau Regions & Statistics Canada Regions) |  |
| *Northeast* | 2 (10%) |
| *Midwest* | 3 (15%) |
| *South* | 8 (40%) |
| *West* | 6 (30%) |
| *British Columbia* | 1 (5%) |
| Household income ($USD) | 204,162 ± 118,886 |
| Number of household members | 2.2 ± 0.4 |
| Marital status |  |
| *Married, or in a domestic partnership* | 20 (100%) |
| *Other*** | 0 (0%) |
| Education level |  |
| *Less than a bachelor’s degree**** | 0 (0%) |
| *Bachelor's degree (BA, BS)* | 6 (30%) |
| *Master's degree (MA, MS, MEd)* | 9 (45%) |
| *Doctorate or professional degree (JD, MD, DDS, DVM, PhD, EdD)* | 5 (25%) |
| Employment status |  |
| *Employed full time* | 18 (90%) |
| *Unemployed and not currently looking for work* | 1 (5%) |
| *Student* | 1 (5%) |
| *Other***** | 0 (0%) |
| Gravidity (before current pregnancy, if applicable) |  |
| *Nulligravida* | 15 (75%) |
| *Primigravida or multigravida* | 5 (25%) |
| Parity |  |
| *Nulliparous* | 18 (90%) |
| *Primiparous* | 1 (5%) |
| *Multiparous* | 1 (5%) |

*Grouping the following race categories with zero participants: American Indian or Alaskan Native; Asian; Black or African American; Native Hawaiian or Pacific Islander. **Grouping the following marital status categories with zero participants: Single; Widowed; Divorced; Separated. ***Grouping the following education level categories with zero participants: High school degree or equivalent (GED); Some college, no degree; Associate degree (AA, AS). ****Grouping the following employment status categories with zero participants: Employed part time; Unemployed and currently looking for work; Retired; Homemaker; Self-employed; Unable to work.

**Table S2.** Bonferroni post hoc test results for one-way ANOVA (TBW ~ time period) in athletes only.

|  | Difference | 95% CI | p-value |
| --- | --- | --- | --- |
| early-preconception | -1.74 | (-5.90, 2.42) | 0.94 |
| third-preconception | 2.82 | (-1.29, 6.92) | 0.31 |
| third-early | 4.56 | (1.53, 7.60) | 0.002** |
| * p≤0.05, **p<0.01, ***p<0.001 | | | |

**Table S3.** Bonferroni post hoc test results for one-way ANOVA (WT ~ time period) in athletes only.

|  | Difference | 95% CI | p-value |
| --- | --- | --- | --- |
| early-preconception | -0.74 | (-1.98, 0.49) | 0.46 |
| third-preconception | -0.86 | (-2.08, 0.36) | 0.28 |
| third-early | -0.12 | (-1.02, 0.79) | 0.95 |
| * p≤0.05, **p<0.01, ***p<0.001 | | | |

**Table S4.** Bonferroni post hoc test results for one-way ANOVA (running distance ~ time period) in athletes only.

|  | Difference | 95% CI | p-value |
| --- | --- | --- | --- |
| early-preconception | 3.03 | (-19.8, 25.9) | 0.99 |
| third-preconception | -24.6 | (-47.2, -2.1) | 0.03* |
| third-early | -27.7 | (-44.3, -11.0) | 0.0007*** |
| * p≤0.05, **p<0.01, ***p<0.001 | | | |

**Table S5.** Bonferroni post hoc test results for one-way ANOVA (MVPA in 10-min bouts ~ time period) in athletes only.

|  | Difference | 95% CI | p-value |
| --- | --- | --- | --- |
| early-preconception | -51.4 | (-252.4, 149.6) | 0.99 |
| third-preconception | -131.4 | (-329.3, 66.4) | 0.34 |
| third-early | -80.0 | (-229.4, 69.4) | 0.60 |
| * p≤0.05, **p<0.01, ***p<0.001 | | | |

**Table S6.** Bonferroni post hoc test results for one-way ANOVA (step count ~ time period) in athletes only.

|  | Difference | 95% CI | p-value |
| --- | --- | --- | --- |
| early-preconception | -2513.1 | (-6443.0, 1416.9) | 0.38 |
| third-preconception | -4848.9 | (-8723.5, -974.4) | 0.01* |
| third-early | -2335.8 | (-5202.3, 530.6) | 0.16 |
| * p≤0.05, **p<0.01, ***p<0.001 | | | |

**Table S7.** Bonferroni post hoc test results for one-way ANOVA (WT ~ HI) in athletes only.

|  | Difference | 95% CI | p-value |
| --- | --- | --- | --- |
| early-preconception | 5.99 | (14.76, 26.74) | 0.99 |
| third-preconception | 6.17 | (-14.28, 26.63) | 0.99 |
| third-early | 0.18 | (-14.95, 15.32) | 0.99 |
| * p≤0.05, **p<0.01, ***p<0.001 | | | |

**
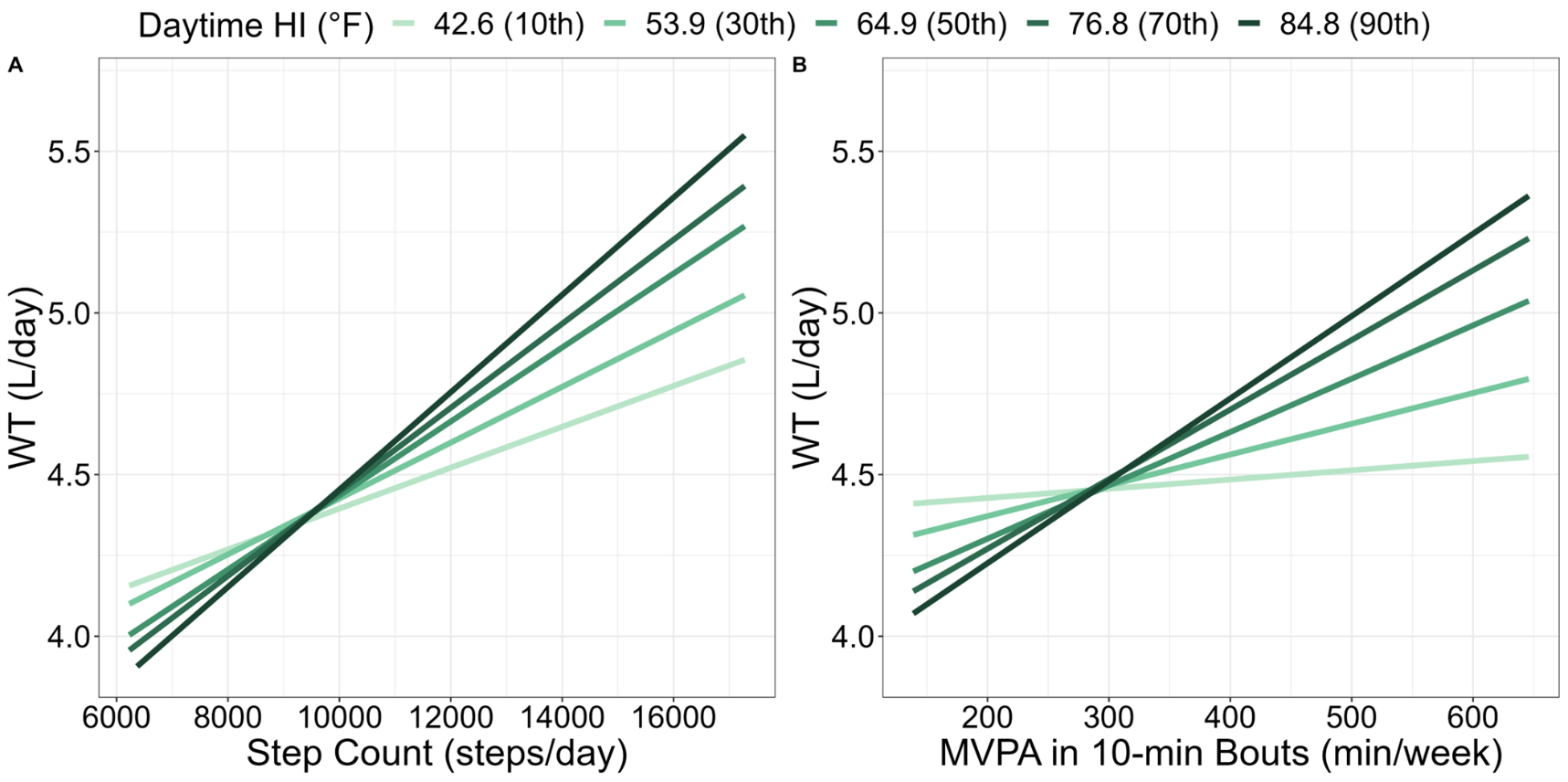
**

**Figure S1.** Post-estimation plots visualizing the impact of PA on WT based on daytime HI levels. (A) Step count positively predicts WT (L/day), with a stronger, more positive slope with higher daytime HI. (B) MVPA in 10-min bouts (min/week) positively predicts WT (L/day), with a stronger, more positive slope with higher daytime HI.

**Table S8.** Linear regression models for the preconception time period in athletes only.

| *WT ~ HI + MVPA + BMI + gestational age* | | | *WT ~ HI + step count + BMI + gestational age* | | |
| --- | --- | --- | --- | --- | --- |
| *Covariate* | *β Coefficient*  *(S.E.)* | *p-value* | *Covariate* | *β Coefficient (S.E.)* | *p-value* |
| (Intercept) | 2.34  (5.38) | 0.69 | (Intercept) | 1.45  (2.77) | 0.64 |
| HI | 0.012  (0.022) | 0.62 | HI | 0.019  (0.011) | 0.20 |
| MVPA | 0.0067  (0.0032) | 0.13 | Step count | 0.00034  (0.000069) | 0.02* |
| BMI | -0.037  (0.22) | 0.88 | BMI | -0.088  (0.11) | 0.49 |
| Multiple R-Squared | 0.65 | | Multiple R-Squared | 0.90 | |
| Adjusted R-Squared | 0.30 | | Adjusted R-Squared | 0.80 | |
| *p≤0.05; **p<0.01; ***p<0.001 | | | | | |

**Table S9.** Linear regression models for the early pregnancy time period in athletes only.

| *WT ~ HI + MVPA + BMI + gestational age* | | | *WT ~ HI + step count + BMI + gestational age* | | |
| --- | --- | --- | --- | --- | --- |
| *Covariate* | *β Coefficient*  *(S.E.)* | *p-value* | *Covariate* | *β Coefficient (S.E.)* | *p-value* |
| (Intercept) | -2.52  (2.86) | 0.40 | (Intercept) | -2.85  (2.44) | 0.26 |
| HI | 0.016  (0.014) | 0.27 | HI | 0.023  (0.012) | 0.07 |
| MVPA | 0.0043  (0.0016) | 0.02* | Step count | 0.00025  (0.000073) | 0.004** |
| BMI | 0.22  (0.13) | 0.13 | BMI | 0.13  (0.092) | 0.18 |
| Gestational age | -0.035  (0.077) | 0.65 | Gestational age | 0.0034  (0.066) | 0.96 |
| Multiple R-Squared | 0.52 | | Multiple R-Squared | 0.59 | |
| Adjusted R-Squared | 0.36 | | Adjusted R-Squared | 0.46 | |
| *p≤0.05; **p<0.01; ***p<0.001 | | | | | |

**Table S10.** Linear regression models for the third trimester time period in athletes only.

| *WT ~ HI + MVPA + BMI + gestational age* | | | *WT ~ HI + step count + BMI + gestational age* | | |
| --- | --- | --- | --- | --- | --- |
| *Covariate* | *β Coefficient*  *(S.E.)* | *p-value* | *Covariate* | *β Coefficient (S.E.)* | *p-value* |
| (Intercept) | 27.05  (13.35) | 0.06 | (Intercept) | 0.29  (0.14) | 0.06 |
| HI | -0.018  (0.012) | 0.14 | HI | -0.019  (0.012) | 0.13 |
| MVPA | 0.0011  (0.0012) | 0.38 | Step count | 0.00005  (0.000062) | 0.43 |
| BMI | -0.017  (0.11) | 0.88 | BMI | -0.082  (0.095) | 0.40 |
| Gestational age | -0.65  (0.35) | 0.08 | Gestational age | -0.66  (0.39) | 0.11 |
| Multiple R-Squared | 0.43 | | Multiple R-Squared | 0.41 | |
| Adjusted R-Squared | 0.27 | | Adjusted R-Squared | 0.25 | |
| *p≤0.05; **p<0.01; ***p<0.001 | | | | | |

**Table S11.** Results for one-way ANOVA (TBW ~ group) and one-way ANCOVA (TBW ~ group + body mass + gestational age) in athletes and non-athletes.

| *WT ~ group* | | | | *WT ~ group + body mass + gestational age* | | | |
| --- | --- | --- | --- | --- | --- | --- | --- |
| *Covariate* | *df* | *F-value* | *p-value* | *Covariate* | *df* | *F-value* | *p-value* |
| (Intercept) | 1 | 4945.13 | <0.0001*** | (Intercept) | 1 | 13.18 | 0.0003*** |
| Group | 1 | 2.87 | 0.09 | Group | 1 | 6.81 | 0.01* |
|  |  |  |  | Body mass | 1 | 239.11 | <0.0001*** |
|  |  |  |  | Gestational age | 1 | 141.61 | <0.0001*** |
| *p≤0.05; **p<0.01; ***p<0.001 | | | | | | | |

**Table S12.** Bonferroni post hoc test results for one-way ANOVA (TBW ~ group) in athletes and non-athletes.

|  | Difference | Adjusted p-value |
| --- | --- | --- |
| Preconception  (athletes – non-athletes) | 3.59 | p<0.0001*** |
| Early pregnancy  (athletes – non-athletes) | -0.45 | P=0.48 |
| Third trimester  (athletes – non-athletes) | -0.62 | p=0.36 |
| * p≤0.05, **p<0.01, ***p<0.001 | | |

**Table S13.** Results for one-way ANOVA (WT ~ group) and one-way ANCOVA (WT ~ group + body mass + gestational age) in athletes and non-athletes.

| *WT ~ group* | | | | *WT ~ group + body mass + gestational age* | | | |
| --- | --- | --- | --- | --- | --- | --- | --- |
| *Covariate* | *df* | *F-value* | *p-value* | *Covariate* | *df* | *F-value* | *p-value* |
| (Intercept) | 1 | 1927.92 | <0.0001*** | (Intercept) | 1 | 12.34 | 0.0005*** |
| Group | 1 | 27.29 | <0.0001*** | Group | 1 | 17.54 | <0.0001*** |
|  |  |  |  | Body mass | 1 | 3.66 | 0.06 |
|  |  |  |  | Gestational age | 1 | 3.35 | 0.07 |
| *p≤0.05; **p<0.01; ***p<0.001 | | | | | | | |

**Table S14.** Bonferroni post hoc test results for one-way ANOVA (WT ~ group) in athletes and non-athletes.

|  | Difference | Adjusted p-value |
| --- | --- | --- |
| Preconception  (athletes – non-athletes) | 1.67 | p<0.0001*** |
| Early pregnancy  (athletes – non-athletes) | 0.73 | p<0.0001*** |
| Third trimester  (athletes – non-athletes) | 0.01 | p=0.99 |
| * p≤0.05, **p<0.01, ***p<0.001 | | |

**REFERENCES**

1. International Atomic Energy Agency. Assessment of Body Composition and Total Energy Expenditure in Humans Using Stable Isotope Techniques. *IAEA Human Health Series* 2009;**3**.

2. Goldberg GR, Prentice AM, Coward WA *et al.* Longitudinal assessment of energy expenditure in pregnancy by the doubly labeled water method. *The American Journal of Clinical Nutrition* 1993;**57**:494–505.

3. Schoeller DA, Fjeld CR. Human Energy Metabolism: What Have We Learned from the Doubly Labeled Water Method? *Annual Review of Nutrition* 1991;**11**:355–73.

4. Singh J, Prentice AM, Diaz E *et al.* Energy expenditure of Gambian women during peak agricultural activity measured by the doubly-labelled water method. *British Journal of Nutrition* 1989;**62**:315–29.

5. Most J, Vallo PM, Altazan AD *et al.* Food Photography Is Not an Accurate Measure of Energy Intake in Obese, Pregnant Women. *The Journal of Nutrition* 2018;**148**:658–63.

6. Svensson Å, Renström F, Bluck L *et al.* Dietary intake assessment in women with different weight and pregnancy status using a short questionnaire. *Public Health Nutrition* 2014;**17**:1939–48.

7. Goldberg GR, Prentice AM, Coward WA *et al.* Longitudinal assessment of the components of energy balance in well-nourished lactating women. *The American Journal of Clinical Nutrition* 1991;**54**:788–98.

8. Jones PJH, Winthrop AL, Schoeller DA *et al.* Validation of Doubly Labeled Water for Assessing Energy Expenditure in Infants. *Pediatric Research* 1987;**21**:242–6.

9. Roberts SB, Coward WA, Ewing G *et al.* Effect of weaning on accuracy of doubly labeled water method in infants. *American Journal of Physiology-Regulatory, Integrative and Comparative Physiology* 1988;**254**:R622–7.

10. Johansson U, Venables M, Öhlund I *et al.* Active Image-Assisted Food Records in Comparison to Regular Food Records: A Validation Study against Doubly Labeled Water in 12-Month-Old Infants. *Nutrients* 2018;**10**:1904.

11. Speakman JR, Yamada Y, Sagayama H *et al.* A standard calculation methodology for human doubly labeled water studies. *Cell Reports Medicine* 2021;**2**:100203.

12. Butte NF, Wong WW, Treuth MS *et al.* Energy requirements during pregnancy based on total energy expenditure and energy deposition. *The American Journal of Clinical Nutrition* 2004;**79**:1078–87.

13. Choi L, Beck C, Liu Z *et al.* Package “PhysicalActivity.” 2021.

14. Migueles JH, Rowlands AV, Huber F *et al.* GGIR: A Research Community–Driven Open Source R Package for Generating Physical Activity and Sleep Outcomes From Multi-Day Raw Accelerometer Data. *Journal for the Measurement of Physical Behaviour* 2019;**2**:188–96.

15. Hildebrand M, Van Hees VT, Hansen BH *et al.* Age Group Comparability of Raw Accelerometer Output from Wrist- and Hip-Worn Monitors. *Medicine & Science in Sports & Exercise* 2014;**46**:1816.

16. Hildebrand M, Hansen BH, van Hees VT *et al.* Evaluation of raw acceleration sedentary thresholds in children and adults. *Scandinavian Journal of Medicine & Science in Sports* 2016;**27**:1814–23.

17. Rowlands AV, Edwardson CL, Davies MJ *et al.* Beyond Cut Points: Accelerometer Metrics that Capture the Physical Activity Profile. *Medicine & Science in Sports & Exercise* 2018;**50**:1323–32.

18. Shapiro SS, Wilk MB. An analysis of variance test for normality (complete samples). *Biometrika* 1965;**52**:591–611.

19. Breusch TS, Pagan AR. A Simple Test for Heteroscedasticity and Random Coefficient Variation. *Econometrica* 1979;**47**:1287–94.

20. Levene H. Robust tests for equality of variances. *Contributions to Probability and Statistics: Essays in Honor of Harold Hotelling*. Stanford University Press, 1960, 278–92.

21. Breusch TS. Testing for autocorrelation in dynamic linear models. *Australian Economics Papers* 1978;**17**:334–55.

22. Godfrey LG. Testing Against General Autoregressive and Moving Average Error Models when the Regressors Include Lagged Dependent Variables. *Econometrica* 1978;**46**:1293–301.

23. Durbin J. Errors in Variables. *Revue de l’Institut International de Statistique / Review of the International Statistical Institute* 1954;**22**:23–32.

24. Wu D-M. Alternative Tests of Independence between Stochastic Regressors and Disturbances. *Econometrica* 1973;**41**:733–50.

25. Hausman JA. Specification Tests in Econometrics. *Econometrica* 1978;**46**:1251–71.

26. Nakamura A, Nakamura M. On the Relationships Among Several Specification Error Tests Presented by Durbin, Wu, and Hausman. *Econometrica* 1981;**49**:1583–8.
